# 17beta-estradiol promotes the odonto/osteogenic differentiation of stem cells from apical papilla via mitogen-activated protein kinase pathway

**DOI:** 10.1186/scrt515

**Published:** 2014-11-17

**Authors:** Yao Li, Ming Yan, Zilu Wang, Yangyu Zheng, Junjun Li, Shu Ma, Genxia Liu, Jinhua Yu

**Affiliations:** Department of Stomatology, Nanjing Integrated Traditional Chinese and Western Medicine Hospital Affiliated with Nanjing University of Chinese Medicine, Nanjing, Jiangsu 210014 China; Key Laboratory of Oral Diseases of Jiangsu Province and, Stomatological Institute of Nanjing Medical University, 140 Hanzhong Road, Nanjing, Jiangsu 210029 China; Endodontic Department, School of Stomatology, Nanjing Medical University, 136 Hanzhong Road, Nanjing, Jiangsu 210029 China

## Abstract

**Introduction:**

Estrogen plays an important role in the osteogenic differentiation of mesenchymal stem cells, while stem cells from apical papilla (SCAP) can contribute to the formation of dentin/bone-like tissues. To date, the effects of estrogen on the differentiation of SCAP remain unclear.

**Methods:**

SCAP was isolated and treated with 10^-7^ M 17beta-estradiol (E2). The odonto/osteogenic potency and the involvement of mitogen-activated protein kinase (MAPK) signaling pathway were subsequently investigated by using methyl-thiazolyl-tetrazolium (MTT) assay, and other methods.

**Results:**

MTT and flow cytometry results demonstrated that E2 treatment had no effect on the proliferation of SCAP *in vitro*, while alkaline phosphatase (ALP) assay and alizarin red staining showed that E2 can significantly promote ALP activity and mineralization ability in SCAP. Real-time reverse transcription polymerase chain reaction (RT-PCR) and western blot assay revealed that the odonto/osteogenic markers (*ALP*, *DMP1*/DMP1, *DSPP*/DSP, *RUNX2*/RUNX2, *OSX*/OSX and *OCN*/OCN) were significantly upregulated in E2-treated SCAP. In addition, the expression of phosphor-p38 and phosphor-JNK in these stem cells was enhanced by E2 treatment, as was the expression of the nuclear downstream transcription factors including phosphor-Sp1, phosphor-Elk-1, phosphor-c-Jun and phosphor-c-Fos, indicating the activation of MAPK signaling pathway during the odonto/osteogenic differentiation of E2-treated SCAP. Conversely, the differentiation of E2-treated SCAP was inhibited in the presence of MAPK specific inhibitors.

**Conclusions:**

The ondonto/osteogenic differentiation of SCAP is enhanced by 10^-7^ M 17beta-estradiol via the activation of MAPK signaling pathway.

## Introduction

When the dental pulp of a young tooth with open apex is infected due to caries or trauma, dentists often try to take measures (for example, apexification and apexogenesis) to preserve the root development and maturation [1]. During the process, a series of factors are involved in the growth of the root, for instance, stem cells from apical papilla (SCAP), which still exist after the formation of the root and can survive the infection [2, 3]. SCAP have a high proliferation rate and possess osteo/dentinogenic and adipogenic potentials [4, 5]. When transplanted into immunocompromised mice, SCAP can form a typical dentin-pulp-like complex and generate bone-like tissues containing osteoblast-like cells [4–6]. Moreover, the combination of SCAP and periodontal ligament stem cells (PDLSCs) can produce dentin and cementum with collagen fibers anchored into the cementum after transplantation *in vivo*, which represents an approach to biological root engineering [7]. Thus, such a biological root supporting a porcelain crown can restore a missing tooth instead of bridges and removable dentures. Huang *et al*. [2] have suggested that SCAP play an important role in root formation. Many factors can affect the proliferation and odonto/osteogenic differentiation of SCAP, including culture medium, cell phenotype, medicaments, growth factors, hormones, morphogens, and so on [8–11] that demonstrate the importance of the extrinsic microenvironment to SCAP when applied in tissue engineering.

Hormones are very important to the growth, development, reproduction and maintenance of a diverse range of mammalian tissues. Estrogen is known as one of the important hormones for sex maturation and bone metabolism [12]. Previous research has revealed a close relationship between estrogen deficiency and osteoporosis that occurs in postmenopausal women, and older men as well, leading to decreased bone mineral density and even bone fracture [13]. It has also been demonstrated that periodontal diseases are related to estrogen deficiency which causes impaired osteogenic differentiation of PDLSCs [14]. Furthermore, an *in vivo* study has shown that the differentiation ability of dental pulp stem cells (DPSCs) was downregulated in estrogen deficient rats [12]. Recent studies have suggested that exogenous estrogen can enhance the proliferation and differentiation of bone marrow mesenchymal stem cells (BMMSCs), PDLSCs and DPSCs [8, 15, 16]. To date, the effects of estrogen on SCAP remain unclear.

In this study, we investigated the influence of estrogen on the proliferation and odonto/osteogenic differentiation of SCAP *in vitro*. SCAP was isolated from extracted third molars and exposed to 17beta-estradiol (E2) [17]. Then, the proliferation, differentiation and involvement of the MAPK signaling pathway in E2-treated SCAP were determined. Our findings suggest that E2 can enhance the odonto/osteogenic differentiation of SCAP via the MAPK pathway.

## Methods

### Cell isolation and culture

Impacted non-carious third molars (n = 20) were collected from young patients (17- to 20-years old) in the Oral Surgery Department of Jiangsu Provincial Stomatological Hospital. This study was approved by the Ethical Committee of the Stomatological School of Nanjing Medical University (Reference #200900128), and consent from patients was obtained. The apical papillae were carefully separated from the immature roots, minced and digested in a solution containing 3 mg/ml collagenase type I (Sigma, St. Louis, MO, USA) and 4 mg/ml dispase (Sigma) for 30 minutes at 37°C. Single cell suspensions were obtained and cells from different patients were mixed. Isolated cells were cultured in alpha minimum essential medium (α-MEM, Gibco, Life Technologies, Grand Island, NY, USA) supplemented with 10% fetal bovine serum (FBS, Hyclone, Logan, UT, USA), 100 U/mL penicillin and 100 mg/mL streptomycin at 37°C in 5% CO_2_. 17beta-estradiol (E2, Ehrenstorfer Gmbh, Augsburg, Germany) was dissolved in absolute ethyl alcohol at 10^-3^ M and stored at -20°C in the dark. In the subsequent experiments, cells were cultured in α-MEM containing E2 (E2 group) or 0.01% (v/v) ethyl alcohol as control (control group). The culture medium was changed every two days. JNK and p38 specific inhibitors SP600125 (Sigma-Aldrich, St. Louis, MO, USA) and SB203580 (Sigma-Aldrich) were dissolved in dimethyl sulfoxide (DMSO, Sigma-Aldrich) at 100 mM and 20 mM, respectively and stored at -20°C in the dark. They were diluted to 20 μM when added to the culture media. Cells at passages two to four were used for the following experiments.

### Cell identification

To determine the origin of the obtained mesenchymal stem cells, isolated cells were immunostained with antibody against STRO-1 (1:200, Novus Biologicals, Littleton, CO, USA) and their surface markers were measured by flow cytometry. Cells were harvested and incubated with fluorochrome-conjugated rabbit anti-human antibodies: CD34-FITC, CD45-PerCP, CD90-PE, CD105-APC, CD146-APC, CD73-PE (Miltenyi, Bergisch Gladbach, Germany) for 20 minutes at room temperature in the dark. Stained cells were washed twice with 0.01 M phosphate buffer solution (PBS) and analyzed by BD FACSCalibur (BD Biosciences, San Jose, CA, USA).

### *In vivo*transplantation

The animal experiments were approved by the Ethical Committee of the Stomatological School of Nanjing Medical University. SCAP (1 × 10^6^) was collected as a pellet in a sterile tube and seeded gently onto absorbable gelatin sponges (AGS, Nanjing Pharmaceuticals, Nanjing, China), which served as carriers. Then, cell pellets were transplanted into the renal capsules of immunodeficient female rats. After *in vivo* culture for eight weeks, the retrieved implants (n = 6) were fixed in 4% polyoxymethylene, decalcified and processed for hematoxylin and eosin (H & E) staining.

### Immunohistochemistry and immunocytochemistry

Immunohistochemical and immunocytochemical analyses of human tissues or human SCAP were performed by the streptavidin-biotin complex method using the primary antibodies (STRO-1, 1:200, Santa Cruz, Dallas, TX, USA; ER-α, 1:100, Abcam, Cambridge, UK) according to the manufacturers’ recommended protocols [18, 19]. The reaction products were developed in 3, 3′-diaminobenzidine solution with hydrogen peroxide and counterstained with hematoxylin.

### MTT assay

SCAP were seeded into 96-well plates (Nunc, Thermo Scientific, Waltham, MA, USA) at a density of 2 × 10^3^ cells/well for 24 hours and starved in a serum-free medium for another 24 hours. Then the medium was changed to complete medium containing E2. At different time points (days 0, 1, 3, 5, 7, 9 and 11), the cells were treated with MTT (3-[4, 5-dimethylthiazol-2-yl]-2, 5-diphenyl-2, 5-tetrazoliumbromide) solution (5 mg/ml; Sigma-Aldrich) and incubated at 37°C for four hours. Then, the solution was removed and 150 μl/well DMSO was added. The absorbance (OD value) was measured at 490 nm with an automatic enzyme-linked immunosorbent assay reader (ELx800, BioTek Instruments Inc., Grand Island, NY, USA). The experiment was repeated three times and MTT results are expressed as the mean ± SD.

### Colony forming assay

SCAP in the control group and the E2 group were seeded into six-well plates (Nunc, USA) at a density of 1 × 10^2^ cells/well for two weeks. Then, the cells were fixed with 4% paraformaldehyde (PFA), stained with crystal violet (Beyotime, Shanghai, China) and photographed. The colonies were visualized under an inverted microscope (Olympus, Hamburg, Germany). Aggregations of more than 50 cells were defined as colonies and then counted. The experiment was repeated three times.

### Flow cytometry for cell cycle

SCAP were plated into 6-cm culture dishes (Nunc, USA), cultured in α-MEM supplemented with 10% FBS until 60% to 70% confluence, and then serum-starved for 24 hours. E2 was added to the culture media of the experimental groups. After three days of incubation, the cells were harvested and fixed with 75% ice-cold ethanol at 4°C for 30 minutes in the dark. DNA content was measured by FAC-Scan flow cytometer (BD Biosciences, San Jose, CA, USA). Cell cycle fractions (G_0_/G_1_, S, and G_2_/M phases) were determined by flow cytometry (FCM). The experiment was repeated three times.

### Alkaline phosphatase (ALP) activity assay and alizarin red staining

SCAP in the control group and the E2 group were seeded into 96-well plates (Nunc, USA) at a density of 2 × 10^3^ cells/well or 24-well plates (Nunc, USA) at a density of 1 × 10^4^ cells/well and cultured in routine media or mineralization-inducing media (MM) containing α-MEM, 10% FBS, 100 U/ml penicillin, 100 μg/ml streptomycin, 100 μM ascorbic acid, 2 mM 2-glycerophosphate and 10 nM dexamethasone. Alkaline phosphatase (ALP) activity assay was performed as previously reported [20] by using an ALP activity kit (Sigma-Aldrich) and normalized to total protein content in the cells at days 5 and 7. At day 14, alizarin red staining was carried out as described before [21] and images were acquired using a scanner. Then, nodule staining was destained by 10% cetylpyridinium chloride (CPC) in 10 mM sodium phosphate for 30 minutes at room temperature. The calcium concentration was determined by measuring the absorbance at 526 nm with a universal microplate reader (BioTek Instruments). This experiment was performed in triplicate and the results are presented as the mean ± SD.

### Real-time reverse transcription polymerase chain reaction (real-time RT-PCR)

Total cell RNA was isolated using TRIzol reagent (Invitrogen, New York, NY, USA) according to the manufacturer’s protocol. The concentration and purity of the RNA samples were determined by the absorbance of RNA at 230, 260 and 280 nm, respectively. The mRNA was reverse-transcribed into cDNA by using a PrimeScript RT Master Mix kit (TaKaRa Biotechnology, Dalian, China). Real-time RT-PCR was performed using a SYBR1 Premix Ex Taq™ kit (TaKaRa, Otsu, Japan) and ABI 7300 real-time PCR system. Real-time RT-PCR reaction conditions were: 95°C for 30 seconds; followed by 40 cycles of 95°C for 5 seconds, 60°C for 31 seconds. Primers used in this experiment are listed in Table 1. *GAPDH* was used as an internal control and the expression of osteo/odontoblast-associated genes (*ALP, DSPP, DMP1, RUNX2, OSX* and *OCN*) was measured by the 2^-ΔΔCt^ method as previously reported. Data are given as the mean ± SD of three independent experiments.

**Table 1:** **Sense and antisense primers for real-time reverse transcription polymerase chain reaction**

Genes	Primers	Sequences (5′-3′)
*ALP*	Forward	GACCTCCTCGGAAGACACTC
	Reverse	TGAAGGGCTTCTTGTCTGTG
*DSPP*	Forward	ATATTGAGGGCTGGAATGGGGA
	Reverse	TTTGTGGCTCCAGCATTGTCA
*DMP*1	Forward	CCCTTGGAGAGCAGTGAGTC
	Reverse	CTCCTTTTCCTGTGCTCCTG
*RUNX*2	Forward	TCTTAGAACAAATTCTGCCCTTT
	Reverse	TGCTTTGGTCTTGAAATCACA
*OSX*	Forward	CCTCCTCAGCTCACCTTCTC
	Reverse	GTTGGGAGCCCAAATAGAAA
*OCN*	Forward	AGCAAAGGTGCAGCCTTTGT
	Reverse	GCGCCTGGGTCTCTTCACT
*GAPDH*	Forward	GAAGGTGAAGGTCGGAGTC
	Reverse	GAGATGGTGATGGGATTTC

### Western blot analysis

To explore the effects of E2 on odonto/osteogenic differentiation of SCAP, SCAP in the control group and the E2 group were cultured for three and seven days, respectively, and then collected. For the evaluation of the MAPK signaling pathway, SCAP was seeded on 6-cm dishes. After 24 hours of incubation, cells were serum-starved for another 24 hours before exposure to E2 for 30 minutes, 60 minutes and 90 minutes, respectively. Cells in different groups were washed twice with cold PBS and lysed in radioimmunoprecipitation assay (RIPA) lysis buffer (Beyotime) containing 1 mM phenylmethylsulfonyl fluoride (PMSF). Cell debris was eliminated by centrifugation at 12,000 rpm for 15 minutes. The cytoplasm protein and nucleoprotein were obtained with a Keygen Kit (Keygen Biotech, Nanjing, China). Protein concentration was measured by the Bradford protein assay. A total of 30 μg protein per lane was loaded onto a 10% SDS-PAGE gel for electrophoresis and then electroblotted (Bio-Rad, Hercules, CA, USA) onto 0.22 μm polyvinylidene fluoride (PVDF) membrane (Millipore, Bedford, MA, USA) at 300 mA for one hour. Membranes were blocked with blocking solution (5% non-fat dried skimmed milk powder, 0.01 M PBS, 0.1% Tween-20 (PBST)) at room temperature for two hours, and subsequently incubated with primary antibodies (DMP1, 1:1000, Abcam; DSP, 1:1000, Santa Cruz; RUNX2, 1:1000, Abcam; OSX, 1:1000, Abcam; OCN, 1:1000, Millipore, Billerica, MA, USA; ERK1/2, 1:1000, Bioworld , St. Louis Park, MN, USA; phosphor-ERK1/2, 1:1000, Bioworld; JNK1/2/3, 1:1000, Bioworld; phosphor-JNK1/2/3, 1:1000, Bioworld; p38, 1:1000, Bioworld; phosphor-p38, 1:1000, Bioworld; Elk-1, 1:1000, Cell Signaling, Danvers, MA, USA; phosphor-Elk-1, 1:1000, Cell Signaling; Sp1, 1:1000, Cell Signaling; phosphor-Sp1, 1:1000, Cell Signaling; c-Jun, 1:1000, Cell Signaling; phosphor-c-Jun, 1:1000, Cell Signaling; c-Fos, 1:1000, Cell Signaling; phosphor-c-Fos, 1:1000, Cell Signaling; ER-α, 1:1000, Abcam; β-ACTIN, 1:1000, Bioworld; H3, 1:1000, Bioworld) overnight at 4°C. β-ACTIN and H3 were, respectively, used as the internal controls. Finally, the membranes were washed with PBST for 10 minutes × 3 followed by incubation in secondary antibodies (1:10,000, Boster, Wuhan, China) for one hour at 37°C, and visualized with ImageQuant LAS4000 system (GE Healthcare, Pittsburgh, PA, USA). The results were quantified with ImageJ software (National Institutes of Health, Bethesda, MD, USA). The experiment was repeated three times.

### Immunofluorescence

E2 treated and untreated SCAP were, respectively, cultured on glass coverslips. After three days culture, cells were washed twice with PBS, fixed in 4% polyoxymethylene for 30 minutes at room temperature, permeabilized with 0.5% Tween 20 for 10 minutes and then blocked with goat serum for 30 minutes at 37°C. After that, cells were incubated with primary antibodies (DSP, 1:50, Santa Cruz; RUNX2, 1:100, Abcam; OCN, 1:100, Millipore) overnight at 4°C. The fluorescence-labeled secondary antibody was added and incubated for one hour at room temperature. Nuclei were stained by 4,6-diamidino-2-phenylindole (DAPI; 1:1,000; Invitrogen) for two minutes. Immunofluorescence was visualized under a microscope.

### Statistics

The quantitative results were expressed as the mean ± SD. Independent samples t test, Chi-square test, one-way analysis of variance (ANOVA) and Tukey’s multiple comparison test were performed with SPSS 17.0 software. *P* values <0.05 were considered to be statistically significant.

## Results

### Identification of SCAP

**Figure 1:**
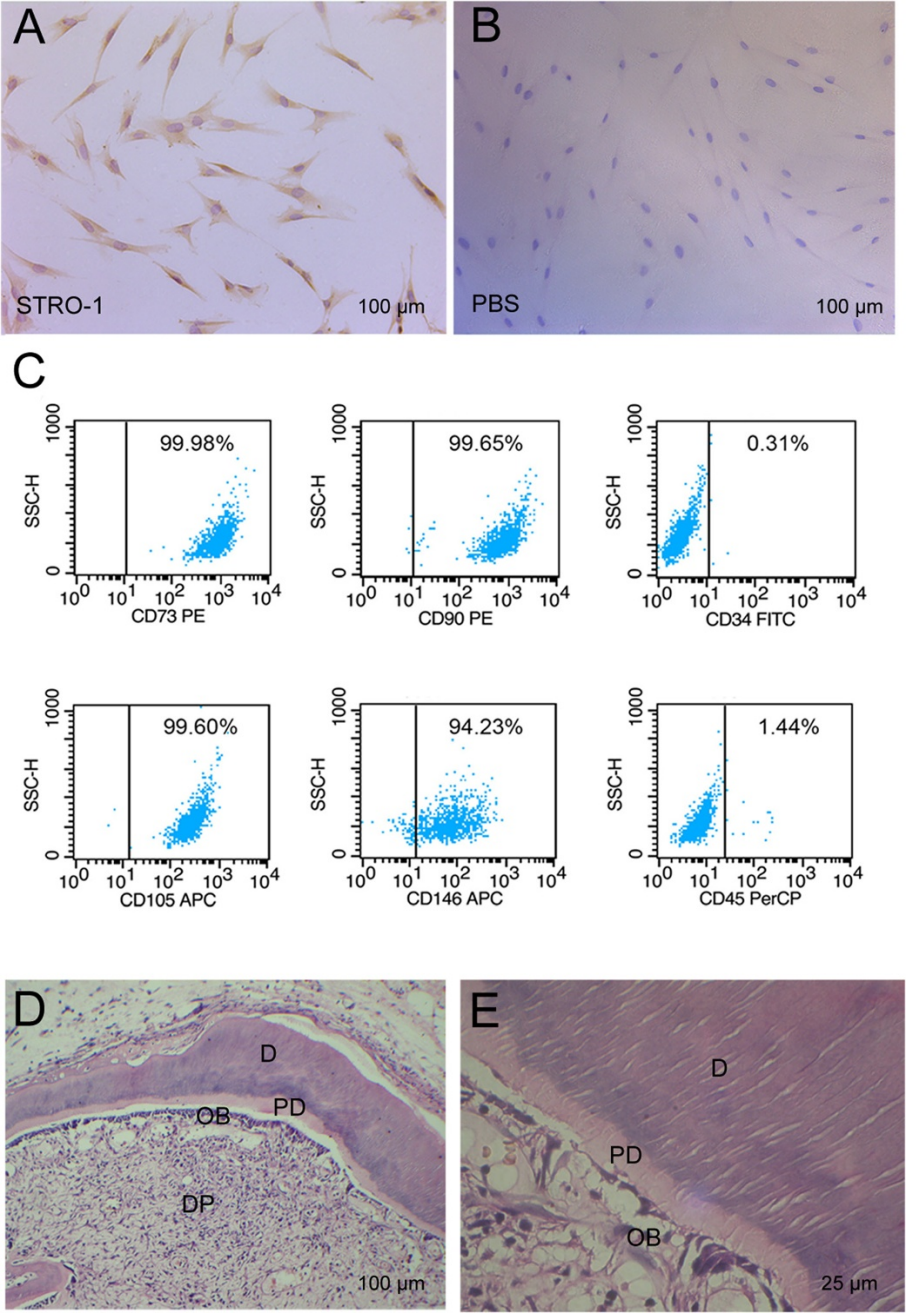
**Identification of SCAP. (A)** Isolated SCAP were stained positively for STRO-1 with typical fibroblast- or spindle-like morphology. **(B)** PBS served as a negative control. **(C)** Isolated SCAP were positive for CD73, CD146, CD90 and CD105, but negative for CD45 and CD34 by flow cytometry. **(D)** *In vivo* transplantation of SCAP pellets formed the typical dentin-pulp-like complex containing dental pulp, odontoblasts, predentin and dentin. **(E)** A higher magnification of **(D)**. DP, dental pulp; OB, odontoblast; D, dentin; PD, predentin. Scale bars: A, B and D = 100 μm, E = 25 μm. SCAP, stem cells from apical papilla.

The isolated cells presented the typical fibroblast- or spindle-like morphology and stained positively for STRO-1 (a mesenchymal stem cell marker, Figure 1A). FCM findings revealed that these cells were positive for CD73, CD146, CD90 and CD105, but negative for CD45 and CD34, indicating that these cells are mesenchymal stem cells (Figure 1C). Moreover, *in vivo* transplantation of such purified cells demonstrated that these cells formed the typical dentin-pulp-like complex containing the structures of dental pulp, odontoblasts, predentin and dentin (Figure 1D). Columnar odontoblasts with polarized nuclei aligned regularly along the predentin (Figure 1E).

### Dose-dependent effects of E2 on SCAP

**Figure 2:**
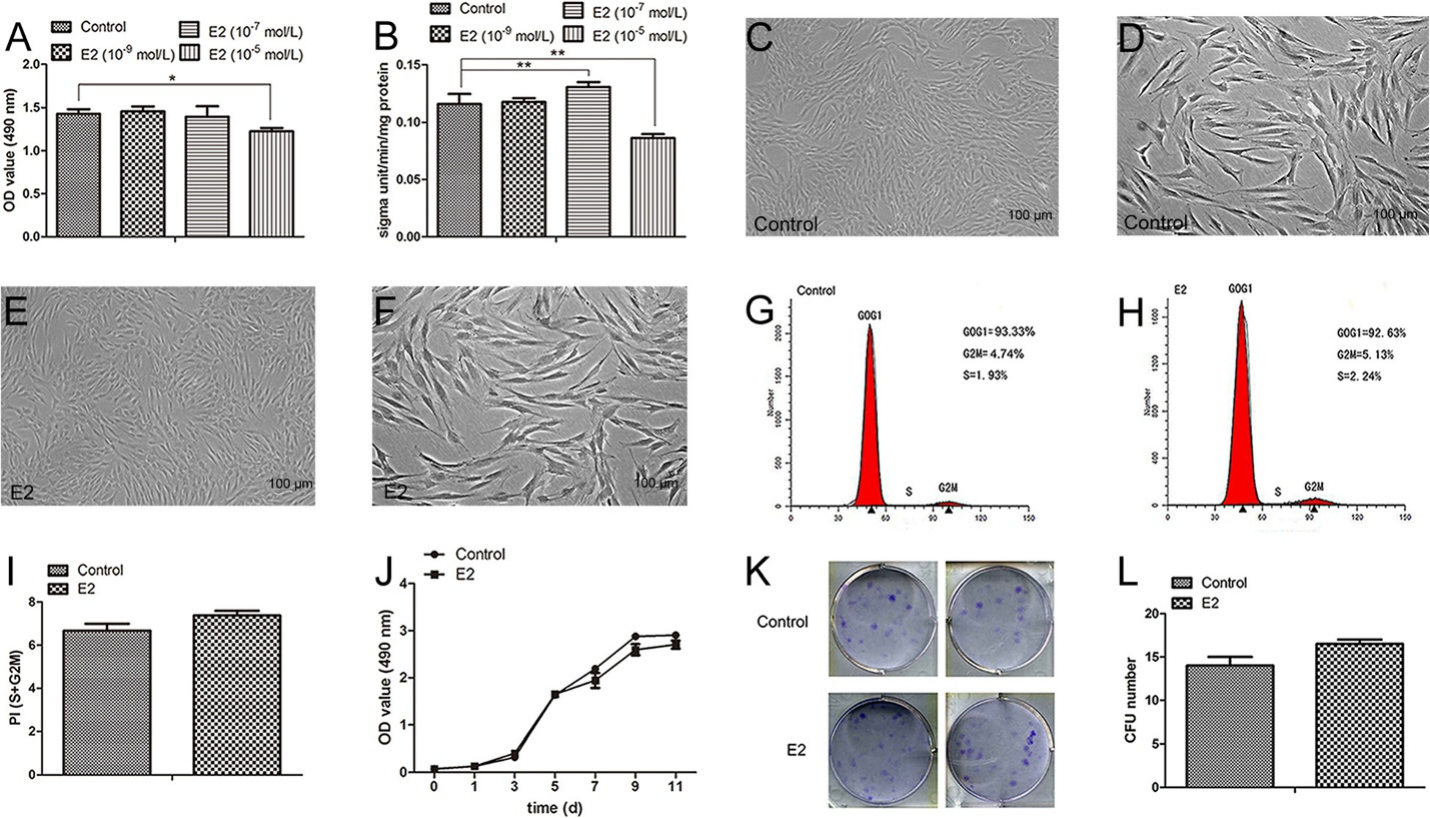
**Effects of E2 on morphology and proliferation of SCAP. (A)** MTT assay at day 5 for E2-treated SCAP at different concentrations (concentration screening). **(B)** ALP activity in E2-treated SCAP at different concentrations (concentration screening). **(C)** The morphology of SCAP in the control group. **(D)** A higher magnification of **(C). (E)** The morphology of SCAP in the E2 group. **(F)** A higher magnification of **(E). (G)** Flow cytometry analysis of control SCAP. **(H)** Flow cytometry analysis of E2-treated SCAP. **(I)** Average proliferation indexes (PI = S + G2M) in the control group (6.67%) and the E2 group (7.37%, *P* >0.05). **(J)** Growth curves for control and E2-treated SCAP. **(K)** Colony forming assay for control and E2-treated SCAP. **(L)** Average number of colonies formed in the control group (14) and the E2 group (16.5, *P* >0.05). Values are given as the mean ± SD, n = 3. *P <0.05, **P <0.01. ALP, alkaline phosphatase; E2, 17beta-estradiol; MTT, methyl-thiazolyl-tetrazolium; SCAP, stem cells from apical papilla; SD, standard deviation.

SCAP were treated with different concentrations of E2 (10^-9^ M, 10^-7^ M, and 10^-5^ M) to detect the dose-dependent effects of E2. MTT results at day 5 demonstrated that 10^-9^ M and 10^-7^ M E2 did not affect the proliferation of SCAP while 10^-5^ M E2 significantly inhibited cell proliferation (Figure 2A; *P* <0.05). ALP activity assay at day 5 revealed that 10^-9^ M E2 had no effect on the differentiation of SCAP while 10^-7^ M E2 significantly promoted cell differentiation (Figure 2B; *P* <0.01). However, the differentiation ability significantly decreased in 10^-5^ M E2-treated SCAP (Figure 2B; *P* <0.01). Thus, 10^-7^ M was selected as the optimal concentration of E2 used in the following experiments.

### Effects of E2 on morphology and proliferation of SCAP

SCAP were cultured in α-MEM containing 10^-7^ M E2 (E2 group) or 0.01% (v/v) ethyl alcohol as control (control group). Both the control group and the E2 group presented a spindle-like morphology and shared almost the same appearance (Figure 2C-F). FCM analysis showed no distinct difference (*P* >0.05) in the proliferation index (PI = G2M + S) between the control group (6.67%) and the E2 group (7.37%) (Figure 2G-I). MTT assay on 11 consecutive days also presented no significant difference (*P* >0.05) between the two groups (Figure 2J), and neither did the results of a colony forming assay between the control group and the E2 group (Figure 2K and L). E2 had no significant effect on the morphology and proliferation of SCAP.

### Effects of E2 on odonto/osteogenic differentiation of SCAP

**Figure 3:**
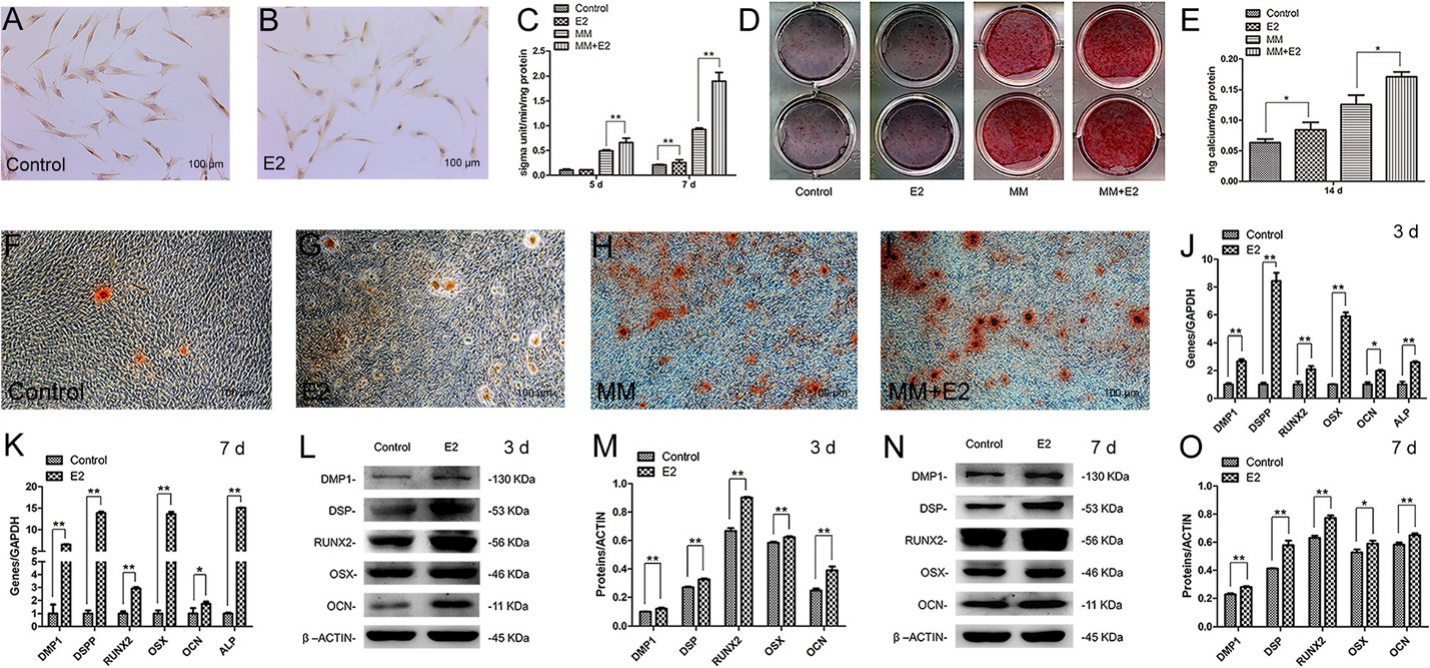
**Odonto/osteogenic differentiation was enhanced in E2-treated SCAP. (A)** Immunocytochemical staining of STRO-1 in the control group. **(B)** Immunocytochemical staining of STRO-1 in the E2 group. Scale bars = 100 μm. **(C)** ALP activity in the control group, E2 group, mineralization-inducing group (MM) and MM + E2 group at day 5 and 7, respectively. Values are presented as the mean ± SD, n = 3. **P <0.01. **(D)** Alizarin red staining for the control group, E2 group, MM group and MM + E2 group at day 14. **(E)** Quantitative analysis of calcium content in the different groups. Values are the mean ± SD, n = 3. *P <0.05. **(F-I)** Calcium nodules in the different groups under the inverted microscope. Scale bars = 100 μm. **(J)** Real-time RT-PCR analysis for the expression of *ALP, DMP1, DSPP, RUNX2, OSX* and *OCN* in control and E2-treated SCAP at day 3. Values are the mean ± SD, n = 3. **2^-ΔΔCt^ >2, *P* <0.01; *1 < 2^-ΔΔCt^ <2, *P* <0.01. **(K)** Real-time RT-PCR analyses for the expression of *ALP, DMP1, DSPP, RUNX2, OSX* and *OCN* in control and E2-treated SCAP at day 7. Values are the mean ± SD, n = 3. **2^-ΔΔCt^ >2, *P* <0.01; *1 < 2^-ΔΔCt^ <2, *P* <0.01. **(L)** Western blot assay for the odonto/osteogenic proteins (DMP1, DSP, RUNX2, OSX and OCN) in control and E2-treated SCAP at day 3. **(M)** Quantitative analysis of western blot results at day 3. Values are the mean ± SD, n = 3. **P <0.01. **(N)** Western blot assay of the odonto/osteogenic proteins in control and E2-treated SCAP at day 7. **(O)** Quantitative analysis of western blot results at day 7. Values are the mean ± SD, n = 3. *P <0.05, **P <0.01. ALP, alkaline phosphatase; E2, 17beta estradiol; SCAP, stem cells from apical papilla; SD, standard deviation.

Immunocytochemical analysis of SCAP showed that the staining of STRO-1 in the cytoplasma of E2-treated SCAP was weaker than that in control group, suggesting the downregulation of STRO-1 following the differentiation of E2-treated SCAP (Figure 3A and B).

SCAP were cultured in routine media or MM with or without E2. At day 5, E2 exerted no influence on ALP activity of SCAP in routine media, while ALP activity was noticeably upregulated by E2 in MM (Figure 3C; *P* <0.01). At day 7, both E2-treated groups exhibited a significant increase in ALP activity of SCAP (Figure 3C; *P* <0.01). Moreover, alizarin red staining and quantitative calcium measurement demonstrated that SCAP treated with E2 produced more calcium nodules after 14 days of incubation (Figure 3D and E; *P* <0.05). When observed under an inverted microscope, the calcium nodules formed in routine media were fewer and smaller, whereas SCAP generated more and larger calcium nodules in MM. Moreover, the shape of the calcium nodules in the MM + E2 group was more regular than in other groups (Figure 3F-I).

Real-time RT-PCR showed that the odonto/osteogenic genes (*ALP, DMP1, DSPP, RUNX2, OSX* and *OCN*) were remarkably enhanced in E2-treated SCAP. In particular, the expression of *ALP, DMP1, DSPP, RUNX2* and *OSX* increased from day 3 to day 7 at a significant extent (Figure 3J and K; *P* <0.01), while the expression of *OCN* rose at a similar level at day 3 and day 7 (Figure 3J and K; *P* <0.05). Western blot findings showed that the expression of the odonto/osteogenic proteins (DMP1, DSP, RUNX2, OSX and OCN) was significantly upregulated in E2-treated SCAP at day 3 and 7, in comparison with cells in the control group (Figure 3L-O; *P* <0.01 or *P* <0.05).

**Figure 4:**
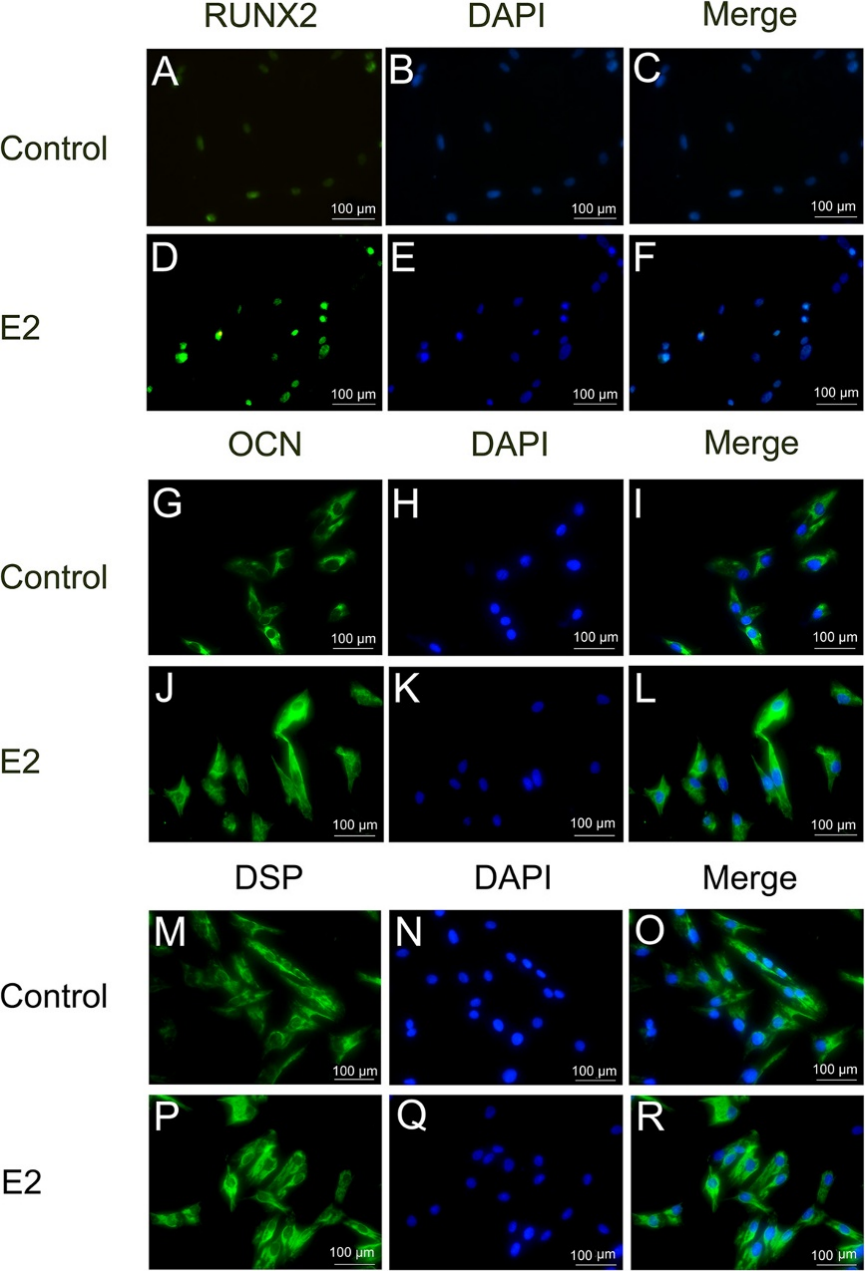
**Immunolocalization of odonto/osteogenic markers in SCAP. (A-C)** Immunofluorescent staining of RUNX2 in control SCAP. **(D-F)** Immunofluorescent staining of RUNX2 in E2-treated SCAP. **(G-I)** Immunofluorescent staining of OCN in control SCAP. **(J-L)** Immunofluorescent staining of OCN in E2-treated SCAP. **(M-O)** Immunofluorescent staining of DSP in control SCAP. **(P-R)** Immunofluorescent staining of DSP in E2-treated SCAP. Scale bars = 100 μm. E2, 17beta estradiol; SCAP, stem cells from apical papilla.

The immunofluorescent results demonstrated that RUNX2 was expressed in the nuclei while OCN and DSP were mainly located in the cytoplasm. They were all enhanced following E2 treatment, indicating the promotion of odonto/osteogenic differentiation in SCAP by E2 (Figure 4).

### Involvement of the MAPK pathway during differentiation of E2-treated SCAP

**Figure 5:**
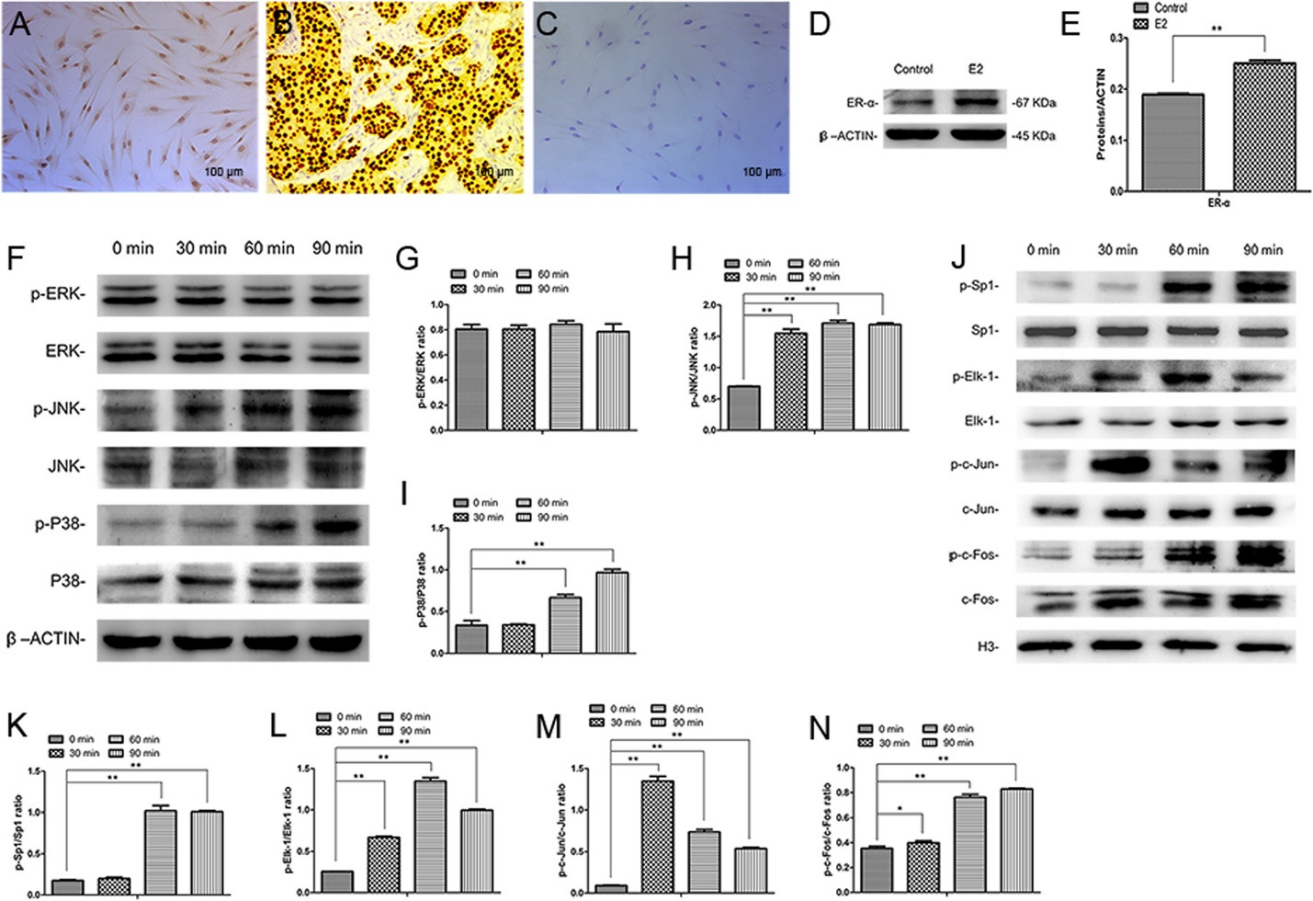
**Activation of the MAPK signaling pathway in E2-mediated odonto/osteogenic differentiation of SCAP. (A)** SCAP were stained positively for ER-α. **(B)** Human breast carcinoma tissue served as a positive control. **(C)** PBS served as a negative control. Scale bars = 100 μm. **(D)** ER-α level detection by western blot analysis in control and E2-treated SCAP. **(E)** Quantitative analysis of the expression of ER-α in control and E2-treated SCAP. Values are mean ± SD, n = 3. **P <0.01. **(F)** The expression of MAPK related proteins (ERK, p-ERK, JNK, p-JNK, p38 and p-p38) by western blot. β-ACTIN was used as the internal control. **(G)** The ratio changes of p-ERK/ERK intensity in E2-treated SCAP. **(H)** The ratio changes of p-JNK/JNK intensity in E2-treated SCAP. **(I)** The ratio changes of p-p38/p38 intensity in E2-treated SCAP. **(J)** The expression of nuclear downstream transcription factors (Sp1, p-Sp1, Elk-1, p-Elk-1, c-Jun, p-c-Jun, c-Fos and p-c-Fos) by western blot analysis. **(K)** The ratio changes of p-Sp1/Sp1 intensity in E2-treated SCAP. **(L)** The ratio changes of p-Elk-1/Elk-1 intensity in E2-treated SCAP. **(M)** The ratio changes of p-c-Jun/c-Jun intensity in E2-treated SCAP. **(N)** The ratio changes of p-c-Fos/c-Fos intensity in E2-treated SCAP. Values are the mean ± SD, n = 3. *P <0.05, **P <0.01. ERα, estrogen receptor α; E2, 17beta estradiol; MAPK, mitogen-activated protein kinase; SCAP, stem cells from apical papilla; SD, standard deviation.

To determine the potential involvement of the MAPK signaling pathway in the E2-mediated differentiation of SCAP, we investigated the ER-α level as well as the expression of MAPK-related proteins and downstream transcription factors. SCAP showed positive staining for ER-α (Figure 5A) and the expression of ER-α was notably enhanced following E2 treatment (Figure 5D and E; *P* <0.01). However, E2 treatment did not affect the expression of total ERK, JNK and p38. The level of phosphor-ERK almost did not change with time, whereas phosphorylation of JNK and p38 gradually upregulated from 0 to 90 minutes following E2 treatment (Figure 5F-I; *P* <0.01). For the nuclear downstream transcription factors, the levels of total Sp1, Elk-1, c-Jun and c-Fos did not change in a time-dependent manner. Phosphorylation of Sp1 was significantly enhanced at 60 minutes and remained at a high level at 90 minutes after E2 treatment (Figure 5J and K; *P* <0.01). Phosphorylation of Elk-1 gradually increased from 0 to 60 minutes but was noticeably downregulated at 90 minutes by E2 treatment (Figure 5J and L; *P* <0.01). In addition, the level of phosphor-c-Jun was significantly upregulated at 30 minutes and then gradually decreased from 60 to 90 minutes following E2 treatment (Figure 5J and M; *P* <0.01). Moreover, the phosphor-c-Fos level rose from 0 to 90 minutes after treatment with E2 (Figure 5J and N; *P* <0.01 or *P* <0.05).

**Figure 6:**
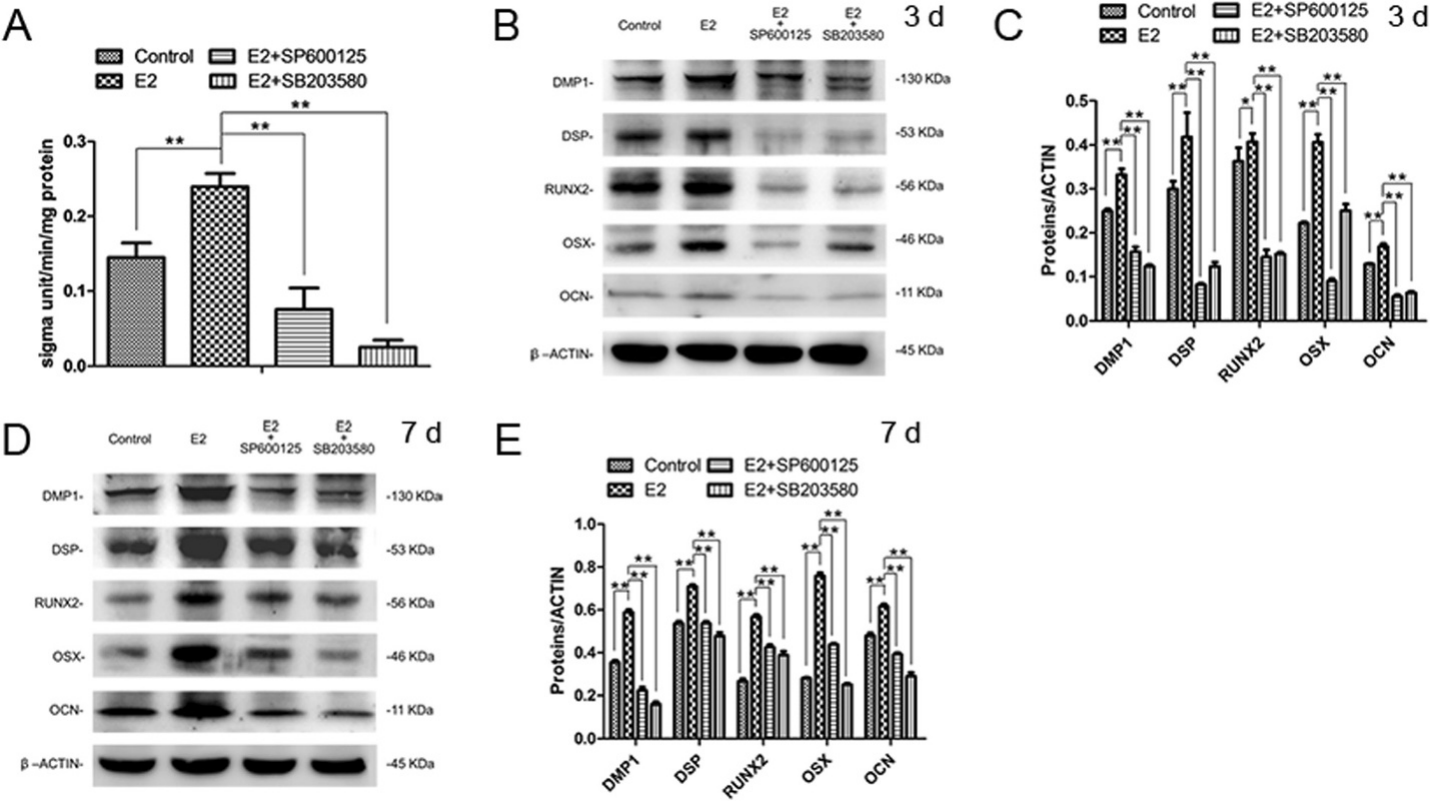
**SP600125 and SB203580 inhibited odonto/osteogenic differentiation of E2-treated SCAP. (A)** ALP activity at day 5 in different groups (control group, E2 group, E2 + SP600125 group and E2 + SB203580 group). **(B)** Odonto/osteogenic protein expression (DMP1, DSP, RUNX2, OSX and OCN) in different groups at day 3. **(C)** Quantitative analysis of odonto/osteogenic proteins (DMP1, DSP, RUNX2, OSX and OCN) in different groups at day 3. **(D)** Western blot analysis for odonto/osteogenic proteins (DMP1, DSP, RUNX2, OSX and OCN) in different groups at day 7. **(E)** Quantitative analysis of odonto/osteogenic proteins (DMP1, DSP, RUNX2, OSX and OCN) in different groups at day 7. Values are the mean ± SD, n = 3. *P <0.05, **P <0.01. ALP, alkaline phosphatase; E2, 17beta-estradiol; SCAP, stem cells from apical papilla; SD, standard deviation.

To further determine the role of the MAPK signaling pathway during the differentiation of E2-treated SCAP, SP600125 and SB203580 (specific inhibitors of JNK and p38) were used at a concentration of 20 μM to suppress the activity of JNK and p38 signaling for one hour prior to E2 treatment. The ALP activity of SCAP at day 5 was remarkably inhibited by SP600125 and SB203580 (Figure 6A; *P* <0.01). Furthermore, odonto/osteogenic proteins (DMP1, DSP, RUNX2, OSX and OCN) were significantly downregulated in the E2 + SP600125 group and the E2 + SB203580 group, in comparison with the E2 group at day 3 and day 7 (Figure 6B-E; *P* <0.01 or *P* <0.05).

## Discussion

Diverse research has revealed that estrogen can exert some effects on bone and mesenchymal stem cells (MSCs). Exogenous estrogen can not only increase bone mineral density and promote bone formation, but also has beneficial effects on the proliferation and differentiation of BMMSCs [16, 22–25]. Even adipose tissue-derived stem cells (ADSCs) can possess osteogenic differentiation potential after estrogen stimulation, with greater nodule formation and mineral deposition [26]. Estrogen has also been reported to increase ALP activity, the expression of osteocalcin and mineralized nodules in PDLSCs [27]. Our previous study revealed that exogenous estrogen at physiological concentrations can enhance the odonto/osteogenic differentiation of DPSCs [8].

In the present study, a higher concentration of E2 (10^-5^ M) significantly inhibited the proliferation of SCAP while 10^-7^ and 10^-9^ M E2 had no effect on cell growth, indicating that there may exist a dose-dependent manner of E2 in regulating the proliferation of SCAP. To evaluate the effects of E2 on the differentiation of SCAP under similar conditions, impacts of E2 on cell proliferation should be excluded. E2 at 10^-7^ M did not have any negative influence on cell morphology and cell proliferation as indicated by MTT, FCM and colony forming assay, while this concentration of E2 can trigger the differentiation of SCAP. Thus, 10^-7^ M E2 was selected as the optimal concentration to stimulate the differentiation of SCAP *in vitro.*

After E2 treatment, ALP activity, mineralization capacity and odonto/osteogenic markers (*ALP*, *DMP1*/DMP1, *DSPP*/DSP, *RUNX2*/RUNX2, *OSX*/OSX and *OCN*/OCN) in SCAP were significantly upregulated. DSP protein and *DSPP* mRNA are tooth-specific markers that contribute to the formation of dentin and they have been reported to be expressed only in secretory odontoblasts [28–30]. DSP protein and *DSPP* mRNA have been identified as late-stage markers of odontoblasts, the expression of which indicates the mature differentiation of odontoblasts [31]. DMP1 is expressed in odontoblasts and secretes matrix proteins to form dentin, which contributes to later dentinogenesis during postnatal development [32]. Moreover, research has suggested that *DSPP* is a downstream effector molecule of *DMP1* which is probably regulated by *DMP1* during dentinogenesis [32, 33]. ALP, RUNX2 and OSX are early-stage markers of osteoblastic differentiation while OCN is related to the late-stage osteoblastic differentiation [24, 34–36]. ALP is expressed early in the developing osteoblast, during the phase of matrix deposition and is downregulated in calcifying osteoblasts [26]. RUNX2 is a crucial factor in osteoblast and odontoblast differentiation, regulating the expression of a variety of bone-/tooth-related genes [29]. Previous studies have revealed that *Runx2*-deficient mice have impaired tooth formation while *RUNX2* overexpression induces new bone generation [37, 38]. *OSX* is a downstream gene of *RUNX2*, which plays an important role in osteogenic differentiation and bone formation and is expressed in functional odonto/osteoblasts [8, 39]. OCN is considered a marker for bone formation in the late stage, which has a close relationship with bone maturation [40]. In the present study, both early-stage and late-stage odonto/osteogenic markers were upregulated, implying the long-term effects of E2 on the differentiation of SCAP.

Previous research has revealed that estrogen stimulates sequential differentiation of osteoblasts and increases calcium deposition in cultured BMSSCs in an estrogen receptor (ER)-dependent manner [41]. As the classic steroid receptors, ERs (including ER-α and ER-β) generally mediate the biological responses to estrogen via genomic and non-genomic effects [42]. It is suggested that the concentration of ERs is an important determinant of cellular responsiveness to estrogen [43–45]. Moreover, an increased level of ER has been detected during the osteoblastic differentiation of rat calvarial osteoblasts [46]. In this study, E2-treated SCAP showed higher expression of ER-α and enhanced odonto/osteogenic potential. Recent studies have suggested that estrogen’s effects on various tissues may be mediated by different cell signaling pathways [47]. E2 can regulate the bioactivity of ER-α-positive endometrial cancer cells on the basis of the MAPK signaling pathway. MAPK are an important family of protein kinases involved in transmitting signals from the cell membrane to the nucleus [48]. There are three well-characterized subfamilies of MAPK, that is, extracellular signal-regulated kinases (ERK), c-Jun N-terminal kinase (JNK) and p38 MAPK. MAPK signaling regulates cell proliferation, differentiation, survival and apoptosis in a variety of cells. In this study, E2 has some effects on the expression of phosphor-JNK and phosphor-p38 but did not change the level of phosphor-ERK, indicating the activation of JNK signaling and p38 MAPK signaling. In addition, the decreased level of mineralization and differentiation in E2-treated SCAP co-cultured with SP600125 or SB203580 confirmed the potential functions of JNK and p38 MAPK signaling. Therefore, it can be concluded that E2 regulates the odonto/osteogenic differentiation of SCAP via activation of the JNK and p38 MAPK pathways.

To further evaluate the MAPK signaling involved in E2-mediated differentiation of SCAP, the nuclear downstream transcription factors of MAPK were investigated by western blot analysis. The phosphorylation of all related transcription factors (phosphor-Sp1, phosphor-Elk-1, phosphor-c-Jun and phosphor-c-Fos) can be detected in a time-dependent manner, indicating the activation of these downstream factors by E2 stimulation. E2 action occurs via the classical genomic and non-genomic ways [49]. The genomic pathway is a slow progress mediated through ERs by binding to an estrogen-responsive element (ERE) in the promotor region of different target genes [50], whereas the non-genomic action of E2 mediated by a membrane-associated receptor (mER) is very fast [51]. It is suggested that liganded ERs can not only bind directly to ERE to regulate the transcription of genes, but also are tethered to DNA by interacting with other transcription factors, such as Sp1 and activating protein-1 (AP-1) to influence the gene expression, which means E2 can induce the expression of related genes through ER/Sp1 and ER/AP-1 complexes [52, 53]. In addition, E2 can trigger rapid effects including activation of a sequential series of kinases and phosphatases through membranes, such as the MAPK signaling pathway, and then induce the phosphorylation of ERs in the nuclei to regulate transcription [42, 54]. As is known, when activated by upstream factors, JNK can stimulate the phosphorylation of c-Jun, its major downstream substrate, which can interact with c-Fos to form a complex, leading to enhanced AP-1 and upregulated transcription activity [55]. In addition, the activation of p38 MAPK also contributes to the formation of AP-1 by producing c-Jun and c-Fos [52]. Moreover, JNK activation can induce the phosphorylation of Elk-1 and promote subsequent transcription activities. Interestingly, E2 activated JNK and p38 MAPK pathways, but had no effect on the ERK pathway in this study. However, the downstream factors of all three of these pathways were activated by E2. This indicates that these downstream transcription factors can not only be activated by their upstream kinases, such as ERK, JNK and p38 MAPK, but can also be triggered by ligand-bound ERs directly. Therefore, we speculate that E2 may play an important role during the crosstalk between the ER and MAPK signaling pathways.

In this study, E2 can promote the odonto/osteogenic differentiation of SCAP. Thus, E2 can be used to stimulate the differentiation of SCAP in the apical papillae suffering from pulp infections to recover the interrupted root development. Moreover, when cultured with PDLSCs together *in vivo*, they can form a biological root (bio-root) as previously reported [7]. Recent studies also reported that E2 can enhance the proliferation and differentiation of PDLSCs [15]. Thus, when the two kinds of cells are co-cultured with E2, they may generate a bio-root more regularly and efficiently. However, tooth growth is a complicated process that is affected by a series of factors, including patients’ conditions, cell activity, growth factors and so on. More work is required to make SCAP and E2 better applied in future clinical practice.

## Conclusions

In conclusion, E2 enhanced the odonto/osteogenic differentiation of SCAP via activation of the MAPK signaling pathway. These findings provide a new insight into the use of E2 for tooth engineering. More work has to be performed to explore other potential mechanisms involved in the differentiation of E2-treated SCAP, which may help the application of E2 in future endodontic practice.

## Abbreviations

ADSCs: adipose tissue-derived stem cells
AGS: absorbable gelatin sponges
ALP: alkaline phosphatase
AP-1: activating protein-1
BMMSCs: bone marrow mesenchymal stem cells
CPC: cetylpyridinium chloride
DMP1: dentin matrix protein 1
DMSO: dimethyl sulfoxide
DPSCs: dental pulp stem cells
DSP: dentin sialoprotein
DSPP: dentin sialophosphoprotein
E2: 17beta-estradiol
ER: estrogen receptor
ERE: estrogen response elements
ERK: extracellular signal-regulated kinase
FBS: fetal bovine serum
FCM: flow cytometry
H & E: hematoxylin and eosin
JNK: c-Jun N-terminal kinase
MAPK: mitogen-activated protein kinase
MM: mineralization-inducing media
MSCs: mesenchymal stem cells
MTT: methyl-thiazolyl-tetrazolium
OCN: osteocalcin
OSX: osterix
PBS: hosphate-buffered solution
PDLSCs: periodontal ligament stem cells
PFA: paraformaldehyde
RT-PCR: reverse transcription polymerase chain reaction
RUNX2: runt-related transcription factor 2
SCAP: stem cells while stem cells from apical papilla
α-MEM: alpha minimum essential medium.

## References

[1] Huang GT (2008). A paradigm shift in endodontic management of immature teeth: conservation of stem cells for regeneration. J Dent.

[2] Huang GT, Sonoyama W, Liu Y, Liu H, Wang S, Shi S (2008). The hidden treasure in apical papilla: the potential role in pulp/dentin regeneration and BioRoot engineering. J Endod.

[3] Huang GT (2009). Apexification: the beginning of its end. Int Endod J.

[4] Sonoyama W, Liu Y, Yamaza T, Tuan RS, Wang S, Shi S, Huang GT (2008). Characterization of the apical papilla and its residing stem cells from human immature permanent teeth: a pilot study. J Endod.

[5] Sonoyama W, Liu Y, Fang D, Yamaza T, Seo BM, Zhang C, Liu H, Gronthos S, Wang CY, Wang S, Shi S (2006). Mesenchymal stem cell-mediated functional tooth regeneration in swine. PLoS One.

[6] Abe S, Yamaguchi S, Watanabe A, Hamada K, Amagasa T (2008). Hard tissue regeneration capacity of apical pulp derived cells (APDCs) from human tooth with immature apex. Biochem Biophys Res Commun.

[7] Dadu SS (2009). Tooth regeneration: current status. Indian J Dent Res.

[8] Wang Y, Zheng Y, Wang Z, Li J, Zhang G, Yu J (2013). 10(–7) m17β-oestradiol enhances odonto/osteogenic potency of human dental pulp stem cells by activation of the NF-κB pathway. Cell Prolif.

[9] Bakopoulou A, Leyhausen G, Volk J, Koidis P, Geurtsen W (2013). Comparative characterization of STRO-1neg/CD146pos and STRO-1pos/CD146pos apical papilla stem cells enriched with flow cytometry. Arch Oral Biol.

[10] Ruparel NB, Teixeira FB, Ferraz CC, Diogenes A (2012). Direct effect of intracanal medicaments on survival of stem cells of the apical papilla. J Endod.

[11] Jiang Q, Du J, Yin X, Shan Z, Ma Y, Ma P, Fan Z (2013). Shh signaling, negatively regulated by BMP signaling, inhibits the osteo/dentinogenic differentiation potentials of mesenchymal stem cells from apical papilla. Mol Cell Biochem.

[12] Wang Y, Yan M, Yu Y, Wu J, Yu J, Fan Z (2013). Estrogen deficiency inhibits the odonto/osteogenic differentiation of dental pulp stem cells via activation of the NF-κB pathway. Cell Tissue Res.

[13] Xue P, Wang Y, Yang J, Li Y (2013). Effects of growth hormone replacement therapy on bone mineral density in growth hormone deficient adults: a meta-analysis. Int J Endocrinol.

[14] Bin Zhang YL, Zhou Q, Ding Y (2011). Estrogen deficiency leads to impaired osteogenic differentiation of periodontal ligament stem cells in rats. Tohoku J Exp Med.

[15] Mamalis A, Markopoulou C, Lagou A, Vrotsos I (2011). Oestrogen regulates proliferation, osteoblastic differentiation, collagen synthesis and periostin gene expression in human periodontal ligament cells through oestrogen receptor beta. Arch Oral Biol.

[16] Zhang M, Chen FM, Wang AH, Chen YJ, Lv X, Wu S, Zhao RN (2012). Estrogen and its receptor enhance mechanobiological effects in compressed bone mesenchymal stem cells. Cells Tissues Organs.

[17] Zhao JW, Gao ZL, Mei H, Li YL, Wang Y (2011). Differentiation of human mesenchymal stem cells: the potential mechanism for estrogen-induced preferential osteoblast versus adipocyte differentiation. Am J Med Sci.

[18] Yu J, Wang Y, Deng Z, Tang L, Li Y, Shi J, Jin Y (2007). Odontogenic capability: bone marrow stromal stem cells versus dental pulp stem cells. Biol Cell.

[19] Lei G, Yan M, Wang Z, Yu Y, Tang C, Yu J, Zhang G (2011). Dentinogenic capacity: immature root papilla stem cells versus mature root pulp stem cells. Biol Cell.

[20] Wang S, Mu J, Fan Z, Yu Y, Yan M, Lei G, Tang C, Wang Z, Zheng Y, Yu J, Zhang G (2012). Insulin-like growth factor 1 can promote the osteogenic differentiation and osteogenesis of stem cells from apical papilla. Stem Cell Res.

[21] Yu J, He H, Tang C, Zhang G, Li Y, Wang R, Shi J, Jin Y (2010). Differentiation potential of STRO-1+ dental pulp stem cells changes during cell passaging. BMC Cell Biol.

[22] Galea GL, Price JS, Lanyon LE (2013). Estrogen receptors’ roles in the control of mechanically adaptive bone (re)modeling. Bonekey Rep.

[23] Ozono S, Fujita T, Matsuo M, Todoki K, Ohtomo T, Negishi H, Kawase T (2008). Co-treatment with basic fibroblast growth factor and 17beta-estradiol in the presence of dexamethasone accelerates bone formation by rat bone marrow stromal cell culture. Nihon Hotetsu Shika Gakkai Zasshi.

[24] Gopalakrishnan V, Vignesh RC, Arunakaran J, Aruldhas MM, Srinivasan N (2006). Effects of glucose and its modulation by insulin and estradiol on BMSC differentiation into osteoblastic lineages. Biochem Cell Biol.

[25] Hong L, Colpan A, Peptan IA (2006). Modulations of 17-β estradiol on osteogenic and adipogenic differentiations of human mesenchymal stem cells. Tissue Eng.

[26] Taskiran D, Evren V (2011). Stimulatory effect of 17β-estradiol on osteogenic differentiation potential of rat adipose tissue-derived stem cells. Gen Physiol Biophys.

[27] Liang L, Yu JF, Wang Y, Wang G, Ding Y (2008). Effect of estrogen receptor beta on the osteoblastic differentiation function of human periodontal ligament cells. Arch Oral Biol.

[28] Iejima D, Sumita Y, Kagami H, Ando Y, Ueda M (2007). Odontoblast marker gene expression is enhanced by a CC-chemokine family protein MIP-3α in human mesenchymal stem cells. Arch Oral Biol.

[29] Chen S, Gluhak-Heinrich J, Wang YH, Wu YM, Chuang HH, Chen L, Yuan GH, Dong J, Gay I, MacDougall M (2009). Runx2, osx, and dspp in tooth development. J Dent Res.

[30] Yamazaki H, Kunisada T, Miyamoto A, Tagaya H, Hayashi SI (1999). Tooth-specific expression conferred by the regulatory sequences of rat dentin sialoprotein gene in transgenic mice. Biochem Biophys Res Commun.

[31] Yamakoshi Y (2009). Dentinogenesis and Dentin Sialophosphoprotein (DSPP). J Oral Biosci.

[32] Ye L, MacDougall M, Zhang S, Xie Y, Zhang J, Li Z, Lu Y, Mishina Y, Feng JQ (2004). Deletion of dentin matrix protein-1 leads to a partial failure of maturation of predentin into dentin, hypomineralization, and expanded cavities of pulp and root canal during postnatal tooth development. J Biol Chem.

[33] Gibson MP, Zhu Q, Wang S, Liu Q, Liu Y, Wang X, Yuan B, Ruest LB, Feng JQ, D'Souza RN, Qin C, Lu Y (2013). The rescue of dentin matrix protein 1 (DMP1)-deficient tooth defects by the transgenic expression of dentin sialophosphoprotein (DSPP) indicates that DSPP is a downstream effector molecule of DMP1 in dentinogenesis. J Biol Chem.

[34] Komori T (2010). Regulation of osteoblast differentiation by Runx2. Adv Exp Med Biol.

[35] Ni P, Fu S, Fan M, Guo G, Shi S, Peng J, Luo F, Qian Z (2011). Preparation of poly(ethylene glycol)/polylactide hybrid fibrous scaffolds for bone tissue engineering. Int J Nanomedicine.

[36] Wade-Gueye NM, Boudiffa M, Vanden-Bossche A, Laroche N, Aubin JE, Vico L, Lafage-Proust MH, Malaval L (2012). Absence of bone sialoprotein (BSP) impairs primary bone formation and resorption: the marrow ablation model under PTH challenge. Bone.

[37] D'Souza RN, Aberg T, Gaikwad J, Cavender A, Owen M, Karsenty G, Thesleff I (1999). Cbfa1 is required for epithelial-mesenchymal interactions regulating tooth development in mice. Development.

[38] Takahashi T (2011). Overexpression of Runx2 and MKP-1 stimulates transdifferentiation of 3 T3-L1 preadipocytes into bone-forming osteoblasts in vitro. Calcif Tissue Int.

[39] Baek WY, Lee MA, Jung JW, Kim SY, Akiyama H, de Crombrugghe B, Kim JE (2009). Positive regulation of adult bone formation by osteoblast-specific transcription factor osterix. J Bone Miner Res.

[40] Neugebauer BM, Moore MA, Broess M, Gerstenfeld LC, Hauschka PV (1995). Characterization of structural sequences in the chicken osteocalcin gene: expression of osteocalcin by maturing osteoblasts and by hypertrophic chondrocytes in vitro. J Bone Miner Res.

[41] Qu Q, Perala-Heape M, Kapanen A, Dahllund J, Salo J, Vaananen HK, Harkonen P (1998). Estrogen enhances differentiation of osteoblasts in mouse bone marrow culture. Bone.

[42] Heldring N, Pike A, Andersson S, Matthews J, Cheng G, Hartman J, Tujague M, Strom A, Treuter E, Warner M, Gustafsson JA (2007). Estrogen receptors: how do they signal and what are their targets. Physiol Rev.

[43] Kuiper GG, Enmark E, Pelto-Huikko M, Nilsson S, Gustafsson JA (1996). Cloning of a novel receptor expressed in rat prostate and ovary. Proc Natl Acad Sci U S A.

[44] Tremblay GB, Tremblay A, Copeland NG, Gilbert DJ, Jenkins NA, Labrie F, Giguere V (1997). Cloning, chromosomal localization, and functional analysis of the murine estrogen receptor beta. Mol Endocrinol.

[45] Santagati S, Gianazza E, Agrati P, Vegeto E, Patrone C, Pollio G, Maggi A (1997). Oligonucleotide squelching reveals the mechanism of estrogen receptor autologous down-regulation. Mol Endocrinol.

[46] Bodine PV, Henderson RA, Green J, Aronow M, Owen T, Stein GS, Lian JB, Komm BS (1998). Estrogen receptor-alpha is developmentally regulated during osteoblast differentiation and contributes to selective responsiveness of gene expression. Endocrinology.

[47] Kousteni S, Han L, Chen JR, Almeida M, Plotkin LI, Bellido T, Manolagas SC (2003). Kinase-mediated regulation of common transcription factors accounts for the bone-protective effects of sex steroids. J Clin Invest.

[48] Migliaccio A, Zhou C, Steplowski TA, Dickens HK, Malloy KM, Gehrig PA, Boggess JF, Bae-Jump VL (2013). Estrogen induction of telomerase activity through regulation of the mitogen-activated protein kinase (MAPK) dependent pathway in human endometrial cancer cells. PLoS One.

[49] Derwahl M, Nicula D (2014). Estrogen and its role in thyroid cancer. Endocr Relat Cancer.

[50] Nilsson S, Makela S, Treuter E, Tujague M, Thomsen J, Andersson G, Enmark E, Pettersson K, Warner M, Gustafsson JA (2001). Mechanisms of estrogen action. Physiol Rev.

[51] Moriarty K, Kim KH, Bender JR (2006). Minireview: estrogen receptor-mediated rapid signaling. Endocrinology.

[52] Saville B, Wormke M, Wang F, Nguyen T, Enmark E, Kuiper G, Gustafsson JA, Safe S (2000). Ligand-, cell-, and estrogen receptor subtype (alpha /beta )-dependent activation at GC-rich (Sp1) promoter elements. J Biol Chem.

[53] Babu RL, Naveen Kumar M, Patil RH, Devaraju KS, Ramesh GT, Sharma SC (2013). Effect of estrogen and tamoxifen on the expression pattern of AP-1 factors in MCF-7 cells: role of c-Jun, c-Fos, and Fra-1 in cell cycle regulation. Mol Cell Biochem.

[54] Xiao HH, Gao QG, Zhang Y, Wong KC, Dai Y, Yao XS, Wong MS (2014). Vanillic acid exerts oestrogen-like activities in osteoblast-like UMR 106 cells through MAP kinase (MEK/ERK)-mediated ER signaling pathway. J Steroid Biochem Mol Biol.

[55] Tang YQ, Jaganath I, Manikam R, Sekaran SD (2013). Phyllanthus suppresses prostate cancer cell, PC-3, proliferation and induces apoptosis through multiple signalling pathways (MAPKs, PI3K/Akt, NFkappaB, and hypoxia). Evid Based Compl Alternat Med.

